# Safe-and-Sustainable-by-Design Framework Based on a Prospective Life Cycle Assessment: Lessons Learned from a Nano-Titanium Dioxide Case Study

**DOI:** 10.3390/ijerph19074241

**Published:** 2022-04-02

**Authors:** Georgios Archimidis Tsalidis, Lya G. Soeteman-Hernández, Cornelle W. Noorlander, Saeed Saedy, J. Ruud van Ommen, Martina G. Vijver, Gijsbert Korevaar

**Affiliations:** 1 Engineering Systems and Services Department, Faculty of Technology, Policy and Management, Delft University of Technology, 2628 BX Delft, The Netherlands; g.korevaar@tudelft.nl; 2 Department of Biotechnology, Applied Sciences Faculty, Delft University of Technology, 92629 HZ Delft, The Netherlands; 3 Netherlands National Institute for Public Health and the Environment (RIVM), 3720 BA Bilthoven, The Netherlands; lya.hernandez@rivm.nl (L.G.S.-H.); cornelle.noorlander@rivm.nl (C.W.N.); 4 Chemical Engineering Department, Applied Sciences Faculty, Delft University of Technology, 2629 HZ Delft, The Netherlands; s.saedy@tudelft.nl (S.S.); j.r.vanommen@tudelft.nl (J.R.v.O.); 5 Institute of Environmental Sciences, Faculty of Science, Leiden University, 2333 CC Leiden, The Netherlands; vijver@cml.leidenuniv.nl

**Keywords:** toxic-free environment, nanomaterial, life cycle impact assessment, titanium dioxide nanomaterial, R&D developers, green deal, technological innovations, P25-TiO_2_

## Abstract

Safe-and-sustainable-by-design (SSbD) is a concept that takes a systems approach by integrating safety, sustainability, and functionality throughout a product’s the life cycle. This paper proposes a framework based on a prospective life cycle assessment for early safety and sustainability assessment. The framework’s purpose is to identify environmental sustainability and toxicity hotspots early in the innovation process for future SSbD applicability. If this is impossible, key performance indicators are assessed. Environmental sustainability aspects, such as global warming potential (GWP) and cumulative energy demand (CED), and toxicity aspects, such as human toxicity potential and freshwater ecotoxicity potential, were assessed upon applying the framework on a case study. The case study regarded using nano-titanium dioxide (P25-TiO_2_) or a modified nano-coated version (Cu_2_O-coated/P25-TiO_2_) as photocatalysts to produce hydrogen from water using sunlight. Although there was a decrease in environmental impact (GWP and CED), the modified nano-coated version had a relatively higher level of human toxicity and freshwater eco-toxicity. For the presented case study, SSbD alternatives need to be considered that improve the photocatalytic activity but are not toxic to the environment. This case study illustrates the importance of performing an early safety and environmental sustainability assessment to avoid the development of toxic alternatives.

## 1. Introduction

Europe is moving towards a more sustainable world, with ambitions such as no net emissions of greenhouse gases by 2050 [1], the European Green Deal [2], the circular economy [3], and moving towards a toxic-free environment. In order to meet these policy ambitions, novel tools and strategies are needed, as well as synergies between the different strategies. Designing for safety has a long tradition in several engineering disciplines, and recently this strategy was applied to deal with the uncertainties associated with emerging technologies, such as nanotechnology. The safe-by-design (SbD) concept refers to identifying the risks and uncertainties concerning humans and the environment at an early phase of the innovation process so as to minimize uncertainties, potential hazard(s), and/or exposure. The SbD approach addresses the safety of the material/product and associated processes throughout the whole life cycle: from the research and development phase to production, use, recycling, and disposal [4,5]. SbD is an important strategy for achieving policy ambitions, such as the European Green Deal [2], where SbD is proposed as one of the strategies to help achieving the related goals, such as creating a circular economy (EU Action Plan for Circular Economy [3]) and moving towards a pollution-free environment. In addition, SbD is an important strategy in the Horizon Europe European Partnership on Assessment of Risk of Chemicals and EU Chemical Strategy for Sustainability [6]. Sustainable-by-design is currently in a more exploratory phase, where a first description of the concept can be found in the Horizon Europe research programme: the “Sustainable-by-design” concept takes a systems approach by integrating the safety, circularity, and functionality of advanced materials, products, and processes throughout their life cycle. This concept can be defined as a pre-market approach that focuses on providing a function (or service), while avoiding properties that may be harmful to human health or the environment from a life cycle perspective (see p. 111 of [7]). The European Commission DG Research and Innovations states that there is an urgent need to move from SbD towards safe-and-sustainable-by-design (SSbD) [8]. In order to move towards SSbD, methods are needed to integrate safety and sustainability aspects for SSbD applicability. This paper deals with the use of nanomaterials (NMs) in everyday life and the uncertainty of nano-related toxicity during the entire NM life cycle. For this, methods are needed to gain insight into the uncertainty of NMs and to reduce the uncertainty where possible. In this study, we developed and applied a stepwise framework for prospective life cycle assessment (LCA), focusing on integrating the toxicity of NMs with environmental sustainability aspects.

Sustainability consists of three aspects: the environmental, economic, and social. LCA is a holistic framework [9,10] which is considered to be the most powerful tool for assessing environmental performance. In recent years, significant advancements to this framework have occurred to translate its applicability to emerging technologies and products, including NMs. Thus, an ex-ante or prospective approach was developed to assess technologies and products before they reach market level [11]. Such an approach provides an early assessment of environmental performance when little information is available but greater and more inexpensive opportunities exist for developers to avoid investing in technologies and products with high environmental burdens [12]. LCA studies have focused on developing guidelines for performing ex-ante/prospective LCAs [12,13,14,15], assessing the effect of ex-ante data on LCA results [12,13,14,15,16,17,18] or comparing LCA results during different stages of the innovation process (lab scale, pilot scale, and industrial scale) [16,17,19].

In particular, applying LCA to NMs resulted in several challenges: (a) a lack of foreground empirical data that resulted in being not able to give a full description of the system that will enter the market, (b) a lack of background inventory data to cover all stages of the life cycle (data gaps in LC Inventory) [20,21], and (c) a lack of appropriate characterization factors for nano-relevant environmental impacts (data gaps in life cycle impact assessment (LCIA)) [21].

Data gaps in LCIAs are under significant research investigation because transformation processes and residence time of NMs are difficult to predict [22] and existing multi-media models for chemicals cannot be employed for NMs. Researchers have developed multi-media models for NMs, such as SimpleBox4nano [23], to calculate characterization factors for the most common nanomaterials, such as P25-TiO_2_ [24,25], carbon nanotubes [26,27,28], nano silver [29], and CuO-NMs [22]. Nevertheless, data gaps in life cycle inventories exist mainly due to a lack of knowledge on the environmental releases of NMs during the various life cycle stages.

Recent reviews by Windsor et al. [30] and Glisovic et al. [31] focused on tools for sustainability assessments of NMs. These authors mention that LCAs are often performed on the material itself or on the technologies to make the NMs, but not on the use of NMs, because large-scale applications and the long-term effects of NMs are often unknown [21]. Glisovic et al. [31] focused on the applicability of LCA to emerging technologies and the kind of information that is needed to perform an LCA study of NMs. Table 1 shows an overview of identified studies which aimed to apply LCA, perform a sustainability assessment, or assess the safety of products, including nano-products. However, despite on-going research, a stepwise and detailed framework that can be used by NM developers to guide them in assessing the prospective environmental performance is missing.

This study provides a framework to integrate environmental sustainability aspects with toxicity aspects. This framework allows for the identification of environmental sustainability and toxicity hotspots early in the innovation process for the application of SSbD actions in the design phase. This stepwise framework based on the prospective LCA has a tiered data approach, where data alternatives are considered when data are not available using the data of bulk NM or of read-across frameworks in the literature and by using databases. The proposed prospective LCA provides a first indicator of hotspots that can be taken into account in the design of NMs. Because of data scarcity, particularly for toxicological data and in emissions/disposal locations, the results might have a level of uncertainty that can be reduced with data generation. Sustainability and toxicity aspects were derived by applying an LCA in a case study along with the data mapping of uncertainties.

## 2. Materials and Methods

A stepwise framework was developed on the basis of a prospective LCA with a tiered data approach and tested using nano-titanium dioxide nanomaterial (P25-TiO_2_) and a modified nano-coated version (Cu_2_O-coated/P25-TiO_2_) as a potential safer and more sustainable alternative with better photocatalytic activity early in the design stage.

**Figure 1:**
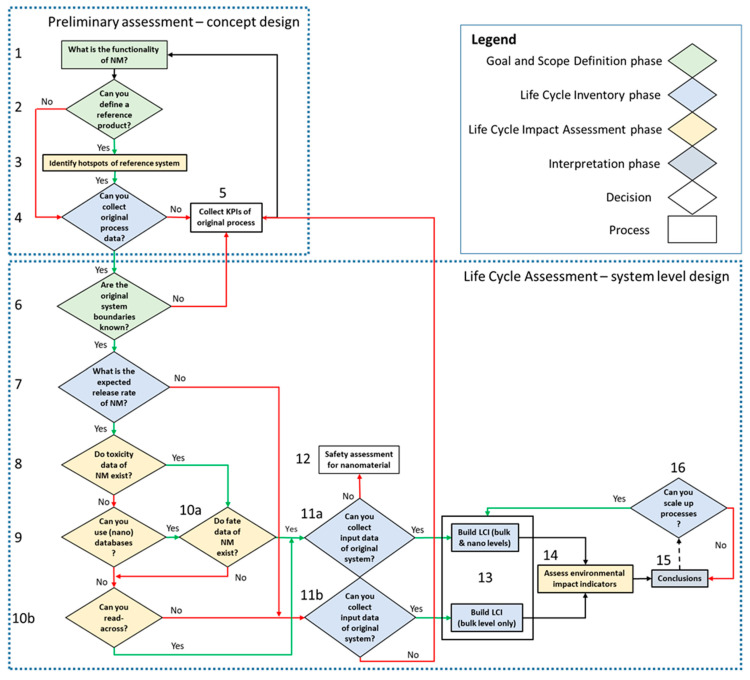
Flow chart of proposed LCA-based framework.

Figure 1 illustrates the framework that was developed and applied to the case study. The framework consists of two parts: (a) a preliminary assessment—the concept design; and (b) a prospective life cycle assessment—the system-level design. Each step of the framework is linked to the four phases of LCA: Phase 1: goal and scope definition; Phase 2: life cycle inventory (LCI) analysis; Phase 3: LCIA; and Phase 4: interpretation of the results [9,10]. This is an iterative process that is used to improve results obtained for the system under study. The prospective LCA-based framework presented in Figure 1 was based mostly on the LCA framework [9,10], and the sustainability environmental impacts measured included global warming potential (GWP) and cumulative energy demand (CED), while the toxicity indicators included human toxicity potential (HTP) and freshwater ecotoxicity potential (FEP).

Preliminary assessment—concept design (Steps 1–5 in Figure 1).

The first part of the framework concerns a preliminary assessment based on conceptual design and serves as a guide to improve prospective environmental performance of the lab-scale process, which concerns NM development, on the basis of KPIs. The first step in the framework is to define the functionality of the NM and to identify a reference product, on the basis of its functionality. The reference product is the single commercial product that offers the same function(s) as the designed product that contains the NM, and against which the designed product is compared. The reference product could have already been considered as a representative for the new product (containing the NM) in the first stages of product development by the product designers. Data are then gathered from databases and/or literature to perform an LCA of the reference system. In case that the new product is a completely new product, and is not similar to any existing product, step 4 is directly performed for the “Preliminary assessment” part of Figure 1. Step 4 concerns finding out if process-level data can be collected regarding the production of the NM. This step is crucial because if process-level data cannot be collected, KPIs based on green chemistry principles [35] should be calculated instead. Table 2 presents an example of the quantitative KPIs used in manufacturing processes. The selection of KPIs depends exclusively on the manufacturing process. Calculated KPIs can be used as input data for the first step and achieve improvements through their optimization. For instance, the goal would be to reduce waste production, energy consumption, operate at atmospheric levels, etc., to simplify the manufacturing stage and reduce its potential footprint.

Prospective Life Cycle Assessment—system-level design (Steps 6–16 in Figure 1).

The second part of the framework concerns a prospective LCA, and its aim is to collect data, to calculate toxicities, and to make suggestions about upscaling. The most important steps are described in Table 3. Step 6, the first of this second part, regards the identification of the original system boundaries where the expected release rate of the NM should be estimated. If this is not possible, then the calculation of KPIs is performed instead. This step is crucial, as it is the first step which addresses explicitly the NM and its toxicity. Therefore, this is the first step of five steps that concern collecting data on the release rate, toxic effect, exposure, and fate via literature, databases, toxicology experiments, reading-across processes, and modelling.

*Step 7 of prospective LCA: What is the expected release rate of NM?*

This step regards the identification of the expected release of NMs to collect data for the following steps.

*Step 8 of prospective LCA: Do toxicological data of the NM exist?*

This step considers the collection of data produced from nano-toxicity studies. The effect Factor (EF), human effect factor (HEF), and exposure factor (XF) are required in this step. Among these factors, XF is linked with the fate factor (FF) mentioned in Step 10. A description of these factors can be found in the Supplementary Materials.

*Step 9 of prospective LCA: Can you use (nano) databases?*

Databases that contain EFs and XFs for specific NMs exist, such as the European Union Observatory for Nanomaterials (https://euon.echa.europa.eu/ (accessed on 15 September 2021)).

Effect factor based on databases: Physicochemical properties should be accounted for and ranked according to which is more relevant to the NM in question. Information can be collected from databases, such as The Nanomaterial Registry (https://nanomaterialregistry.net/ (accessed on 25 March 2022)) and the Nanoparticle Information Library (http://nanoparticlelibrary.net/ (accessed on 25 March 2022)).

Exposure factor based on databases: A common precautionary approach of researchers is to set the XF equal to 1 [24]. Therefore, if calculating the XF is not possible in the previous step, the practitioner is advised to be set it to 1. Later, scenarios can be made in order to refine the calculation of the XF.

*Step 10a of LCA: Do fate data of NM exist?*

This step considers the data available for environmental fate modelling in order to allow for the calculation of the FF. The FF (measured in days) expresses a substance’s residence time in a particular environmental compartment, for instance, freshwater. It is important to realise that during the life cycle of NMs, NMs can change because they encounter other (nano)particles, interact with environmental media, and/or degrade [36]. Multimedia fate models have only recently been applied to estimate environmental background concentrations of NMs [23,37], and the dissolution rate is the most investigated input parameter [38]. These models can be used to investigate NM releases and fate descriptors, such as (hetero-)aggregation, particle sedimentation, re-suspension, wet–dry deposition, ageing processes, agglomeration, corona formation, etc. The main mechanisms that attend to toxicity are: the aggregation and formation of reactive oxygen species [39]. Some recently developed fate modelling tools specifically for NMs during their life cycle are: SimpleBox4Nano [23] and MendNano NMs [40].

*Step 10b of prospective LCA: Can you read across?*

The read-across method for NMs is defined as the use of test data from a toxicity or fate study on one nanoform to cover other nanoforms of the same substance. The application of the read-across method demands the proper characterisation of each nanoform in terms of physicochemical parameters. In addition, data regarding the behaviour and reactivity of each nanoform are required.

*Steps 11a and 11b: Inventory build-up for original system*: In this step, the LCI phase of the LCA framework is performed. Two types of data exist in LCA: background data and foreground data. Background data concern material and energy flows that indirectly affect the LCA system under study, for instance, electricity production in a certain country. All background data should concern bulk materials data. Foreground data concerns processes that are directly connected with the LCA system under study, for instance, the NM production, use, and disposal stages. These processes will be a combination of bulk and nano-material flows, and energy flows.

*Step 12: Safety assessment*: The gathering of toxicity information on NMs early in the innovation phase is challenging due to the lack of available information on newly designed materials. Nevertheless, information on the chemical or pristine NM can be gathered and used as an indicator for the toxicity of the NM. Generally, toxicity information is collected from nano-specific databases, such as eNanoMapper, and data from the bulk materials can be collected from the Classification, Labelling and Packaging inventory in the Registration, Evaluation, Authorisation and Restriction of Chemicals (REACH) from the European Chemical Agency (https://echa.europa.eu/ (accessed on 15 September 2021)) or through public literature.

*Case study: Titanium dioxide with Cu_2_O coating*

**Figure 2:**
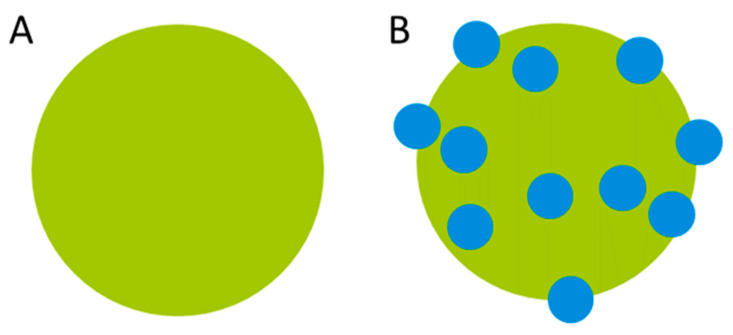
(**A**) P25-TiO_2_ NM; (**B**) P25-TiO_2_ NM decorated (coated) with Cu_2_O NMs.

The case study concerns the improvement of the photocatalytic performance of commercial Evonik P25-TiO_2_ nano-powders in H_2_ production (Figure 2A). The improved photocatalytic alternative was achieved by coating P25-TiO_2_ with Cu_2_O-NMs using the atomic layer deposition method. In this method, both film coating and island growth modes are possible, which depend on the interaction of coated material and substrate. In this particular case, the deposition of Cu_2_O-NMs on TiO_2_ occurred with the island growth mode, which resulted in the TiO_2_ being “decorated” with Cu_2_O-NMs, as shown in Figure 2B. Surface coating is known to significantly influence the (eco)toxicity of the NMs because it affects stability [41]. For instance, surface coating P25-TiO_2_ NMs and anatase P25-TiO_2_ NMs with silica and vanadium pentoxide resulted in increased pulmonary inflammation and a cytotoxicity of up to 400%, respectively, when compared to the same uncoated NMs [42,43,44].

*Goal and scope definition*

**Figure 3:**
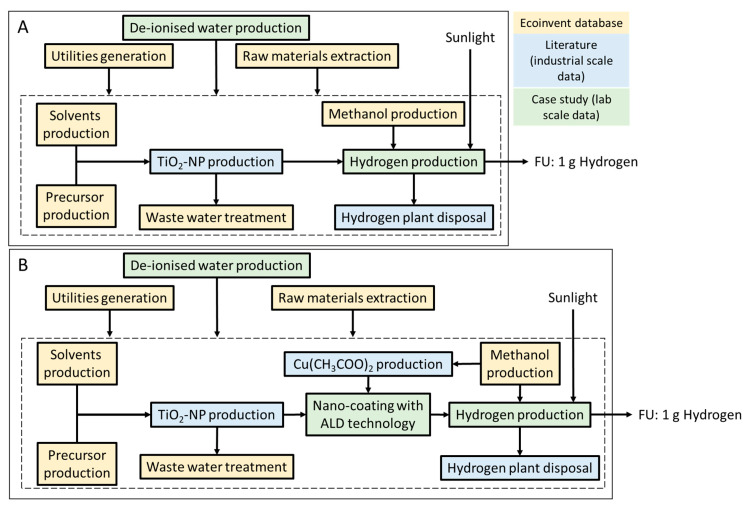
System boundaries of systems (**A**) P25-TiO_2_ and (**B**) Cu_2_O/P25-TiO_2_.

The function of the system is hydrogen production from water using sunlight with the aid of a photocatalyst; that is, a material that strongly enhances a chemical reaction using light as the energy source. Nano-coating P25-TiO_2_ with Cu_2_O aims to improve the photocatalytic performance of the former and increase H_2_ production and might also be a more sustainable alternative to P25-TiO_2_. Therefore, the original system concerns a hydrogen production plant that uses a catalyst based on nano-coated P25-TiO_2_ (system B), while the reference system regards hydrogen production with non-coated P25-TiO_2_ (system A). Nano-coating is performed with a gas-phase coating technology widely used in the semiconductor industry. With this technique, both nanofilms and layers of NMs can be deposited; in this work, we make use of the latter mode. Both hydrogen production plants will operate in a place with abundant sunlight. Figure 3 illustrates the system boundaries of original and reference systems. Since the functionality of the system is hydrogen production, 1 g of H_2_ was selected as functional unit.

*Life cycle inventory*

LCI data are a combination of industrial-scale data and lab-scale data. The developed framework aims to assist product developers. Therefore, its first iteration regards lab-scale data for the production of the nano-coated NMs. The processes in system boundaries where impacts due to nano-size are expected are: the production of P25-TiO_2_ and nano-coated P25-TiO_2_. A detailed LCI is presented in the Supplementary Materials.

*Production of nano P25-titanium dioxide*

Evonik P25-TiO_2_, a TiO_2_ nano-powder, is used in nano-coating and hydrogen production processes because it is suitable for many applications that require high photoactivity. Evonik P25-TiO_2_ is a combination of anatase and rutile forms 80/20 and was purchased from Evonik Industries AG (Hanau, Germany). Evonik Industries AG used the traditional chloride pathway [45] to produce P25, with a mean particle diameter of approximately 21 nm and a specific surface area of approximately 50 m^2^/g. Although P25 consists of NMs, these particles are agglomerated during the production process. This means that nanoparticles are sticking together, and can be separated easily, so the agglomerates can release individual nanoparticles during photocatalysis [46,47].

Copper oxide nano-coating P25–titanium dioxide involves using atomic layer deposition method technology.

Copper oxides (CuO and Cu_2_O) are well-studied metal oxides for photocathodic hydrogen production because they are natural p-types with proper band gaps for light absorption [48]. Nano-coating using the atomic layer deposition method was performed with a home-built fluidized bed reactor [49,50]. P25-TiO_2_ and a high-grade copper(I) hexafluoropentanedionate–vinyltrimethylsilane complex (Cu(I)(hfac)(TMVS)) [51,52,53] were used in the nano-coating process with nitrogen and distilled water [54]. The product was nano-coated Cu_2_O/P25-TiO_2_ and the precursor’s part that did not coat P25-TiO_2_ was burned and released as CO_2_ and water. The Cu(I)(hfac)(TMVS) complex was purchased from ADVANCED TECH. & IND. Co. It was evaporated at 60 °C and deposited on P25-TiO_2_ at 200 °C.

*Hydrogen production*

On the basis of lab-scale experiments, the hydrogen reactor employs water, methanol, and light obtained from a sunlight simulator to produce hydrogen, and the photocatalytic setup is described elsewhere [55]. The catalyst is dispersed in water [34]. Methanol is used as a scavenging agent; however, it also results in further hydrogen generation (CH_3_OH + H_2_O → CO_2_ + 3H_2_) [56].

*Assumptions*

Assumptions were made in order to model both systems. These assumptions are not expected to influence the application of the adapted framework, but they will affect the LCA results. These assumptions are:

(Eco)Toxicology studies are limited regarding Cu_2_O NMs. Therefore, toxicology data for CuO NMs were collected, instead of toxicity data for Cu_2_O NMs.

No data were found regarding the toxicity of P25-TiO_2_ nano-coated with Cu_2_O NMs. Therefore, a read-across method using copper oxide NMs data was used to identify relevant FF and EF in the literature.

We assumed that sunlight will be employed in both systems, even though lab-scale experiments with a photo-reactor employ artificial light.

No production data for Copper(I) hexafluoropentanedionate–vinyltrimethylsilane complex were found. Thus, production data for a similar product, i.e., copper(II) hexafluoroacetylacetonate hydrate, were considered.

No research has been conducted about the expected release of P25-TiO_2_ NMs nano-coated with Cu_2_O. The expected release of P25-TiO_2_ coated with Cu_2_O NMs is similar to the release of P25-TiO_2_, like other metal and metal oxide NMs [57]. Therefore, it is assumed that P25-TiO_2_ NMs nano-coated with Cu_2_O will be released similarly to P25-TiO_2_ NMs because eventually everything will dissolve somewhere in nature and, thus, Cu_2_O/TiO_2_ samples will respond like non-decorated TiO_2_ particles.

Lastly, catalyst recycling was not considered in the system boundaries. These nanoparticles should be recycled and separated in two steps: high-speed centrifuge sedimentation and ultrafiltration. Nanoparticles usually form large agglomerates up to a few micrometres in size, and the agglomerates can easily be recycled/separated using centrifuging. However, the agglomerates break down and individual nanoparticle release is possible. That is why ultrafiltration is recommended to avoid individual nanoparticle release. In a leak-free filtration system, the release of TiO_2_ nanoparticles is not likely.

*Life cycle impact assessment*

LCIA regards four environmental impact indicators: global warming potential (GWP) in kg CO_2_ equivalent, cumulative energy demand (CED) in MJ, human toxicity (non-cancer) potential (HTP) in cases, and freshwater ecotoxicity potential (FEP) in potentially affected species fraction based on Usetox model 2 V1.00 (http://usetox.org/ (accessed on 10 September 2020)). As mentioned before, multimedia models for bulk chemicals cannot be used for NMs and, as a result, toxicity-related results due to nanoforms were calculated manually on the basis of the established framework. GWP and CED impacts were selected because both systems concern the energy conversion and energy transition concepts, while HTP and FEP impacts were selected due to the nano-specific aim of this study. It is expected that nanoforms affect toxicity-related impact indicators [58]. Therefore, we expect GWP and CED to be affected from foreground and background processes, which consist of mostly of electricity, heat, and bulk materials consumption. On the other hand, safety LCA indicators, based on HTP and FEP characterization factors specific to P25-TiO_2_ NMs and P25-TiO_2_ NMs nano-coated with Cu_2_O NMs, need to be calculated or collected.

## 3. Results

### 3.1. Life Cycle Assessment

In this section, data were collected on the basis of the decisions pathway of Figure 1. Therefore, the structure of the framework is used to present the results.

*Steps 1–6*: Earlier steps than Step 7 are described in detail in previous Section 2. The functionality of the original system is hydrogen generation via the nano-coating of the photocatalyst. Therefore, the reference system concerns hydrogen generation without the modification of the P25-TiO_2_ catalyst. Original system data were collected via interaction with the scientists applying atomic layer deposition method on P25-TiO_2_ NMs, and system boundaries were designed on the basis of the considered processes.

*Step 7:* It is important to identify at which life cycle stages NMs will be released. NMs are expected to be released during the NM manufacture process and the nano-coating process to air and sewer water. This is a prospective LCA; therefore, a probabilistic modelling approach needs to be followed for P25-TiO_2_ NMs and P25-TiO_2_ NMs nano-coated with Cu_2_O. Adam et al. [59] calculated the expected release of P25-TiO_2_ NMs during its life cycle. Furthermore, it was assumed that P25-TiO_2_ NMs nano-coated with Cu_2_O will be released similarly to their precursor. No release is expected during operation.

*Step 8:* According to the classification provided by companies to ECHA in REACH registrations, this substance is suspected of causing cancer [60]. Toxicology and fate data exist for P25-TiO_2_ NMs regarding HTP and FEP and for copper oxide NMs concerning FEP. Toxicology and fate data do not exist for nano-coated P25-TiO_2_ NMs with copper oxide NMs.

*Step 9: Databases* were searched thoroughly, but we decided to use data from the literature. Effort was made to create standard operating procedures to test nanomaterials (e.g., EU Patrols) and harmonize databases (e.g., NanoHarmony programmes). However, this will probably take a couple of years before it is openly accessible. Furthermore, toxicology datasets in databases did not exist for Cu_2_O/TiO_2_ when this study was written.

*Step 10a:* Salieri et al. [61] investigated the fate of P25-TiO_2_ in freshwater (see Table 4). As mentioned in Step 7, no fate data exist for nano-coated P25-TiO_2_ with Cu_2_O NMs. We followed the precautionary approach and set it to 1. This means that all species can be exposed to the released NMs.

*Step 10b:* Pu et al. [22,38] investigated the effect and fate factors of copper oxide NMs on the basis of their location.

*Step 11, LCA results:*Table 5 presents the LCA results. Due to the improved efficiency of the photo-reactor, environmental benefits are achieved for GWP and CED. Furthermore, for all bulk material-affected impacts, the dominant factor is methanol production. Methanol is used in hydrogen production processes and functions as a scavenging agent. However, if industrial scale data will be used, then methanol consumption and consequent contribution to GWP will decrease significantly. On the other hand, depending on the selected FF for Cu_2_O, the original system may result in a similar FEP score as for the reference system. Interestingly, HTP and FEP are several orders of magnitude larger for NMs than the same impact potentials for bulk materials. This occurs because low percentages are expected to be released to air, but a considerable amount is expected to be released to surface water due to disposal. This is due to the stronger toxicity of NM when compared with bulk particles of the same origin, e.g., bulk TiO_2_ or P25-TiO_2_.

*Step 12a, safety assessment:* A summary of the preliminary safety assessment is given in this section. Several reports regarding the toxicity of Cu_2_O NMs have been published in recent years, with some studies finding Cu_2_O NMs to be toxic, while others found them to have protective effects against oxidative stress. Leung et al. [62] reported that Cu_2_O NMs samples varying in physicochemical properties exhibited significantly different toxicity. Varying toxicity could likely be attributed to differences in interactions with cells and differences in NMs composition. Particle size is highly important, but also other physicochemical properties, such as surface reactivity and surface shape, may influence the toxicity of nano-copper [63]. Because there is a lack of toxicity studies with Cu_2_O/P25-TiO_2_, data on Cu_2_O NMs were used. Cu_2_O (bulk) and Cu_2_O NMs are toxic to the environment [64]. Additional information that needs to be taken into account is, for instance, the fact that copper-doped P25-TiO_2_ nanotubes produce about five times more OH radicals than undoped TiO_2_ nanotubes and that Cu_2_O/P25-TiO_2_ can be used as effective surface disinfection at low-intensity UVA light of 30 μW/cm^2^, including disinfection against resistant strains, such as methicillin-resistant Staphylococcus aureus (MRSA) and extended-spectrum beta-lactamase Escherichia coli (*E. coli* ESBL) [65]. A full characterization of the Cu_2_O/P25-TiO_2_ is needed in addition to toxicity testing.

### 3.2. Towards an LCA/SSbD Approach

#### 3.2.1. Possible SSbD Actions

This case study was used as a showcase example to highlight the lessons learned when trying to apply SSbD early in the innovation process and using early toxicity analysis and LCA to identify human and environmental hotspots and how to deal with them for future SSbD applicability, as presented in Table 6.

#### 3.2.2. Data Uncertainty

**Figure 4:**
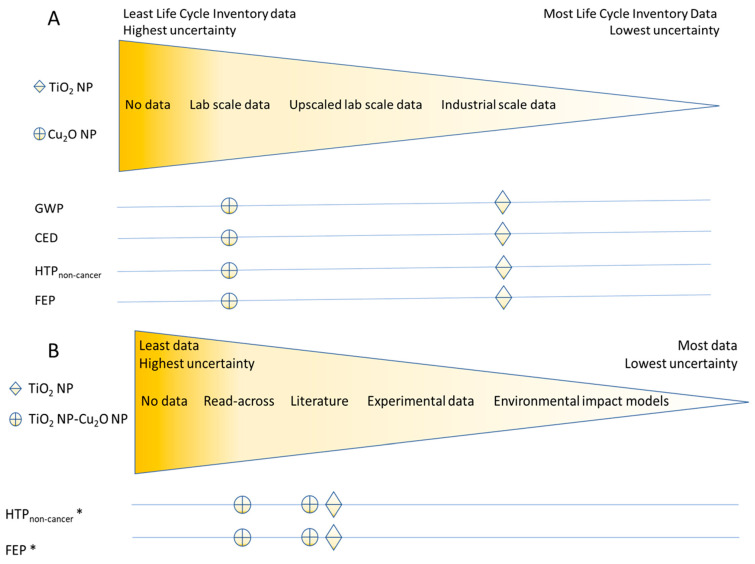
Uncertainty heat map for the different data sources: (**A**) life cycle inventory data scale and environmental impact results; (**B**) data sources for characterization factors and environmental impact results, * environmental impacts due to nano-forms.

Within this prospective LCA framework, we developed two uncertainty heat maps to make uncertainties more transparent (see Figure 4) and guide the user to which nano-specific data need to be collected to decrease uncertainty in the LCA parameters. Figure 4A shows that data for manufacturing and using P25-TiO_2_ NMs and Cu_2_O NMs were collected from lab experiments and the industry. Figure 4B shows that data for calculating characterization factors of HTP and FEP due to nano forms were collected on the basis of the reading-across method and a literature survey.

### 3.3. Lessons Learned

*Contribution of this prospective LCA framework to SSbD applicability*

The prospective LCA presented here can be used for SSbD applicability by identifying possible environmental sustainability and toxicity (safety) hotspots early in the innovation process and, in dialogue with researchers, developing novel materials that are safer and more sustainable without compromising on functionality.

This framework was tested using titanium dioxide nanomaterials (P25-TiO_2_ NMs) and a modified nano-coated version (Cu_2_O-coated/P250TiO_2_) as an alternative with better photocatalytic activity early in the design stage. Although there was a decrease in environmental sustainability impact (GWP and CED), the alternative had a relatively higher level of human and ecotoxicity (HTP and FEP). This case study illustrates the importance of performing an early safety and environmental sustainability assessment. For the presented case study, SSbD alternatives need to be considered that improve the photolytic activity but which are not toxic to the environment.

*Uncertainties can be reduced by generating NM data and by ensuring data generated are FAIR (Findable, Accessible, Interoperable, and Reusable)*

The selection of environmental sustainability impact indicators can be challenging due to data uncertainty. In this study, we selected indicators (GWP and CED) that are relevant to the photocatalytic energy system functionality of the P25-TiO_2_ case study. The GWP, CED, HTP, and FEP data for the P25-TiO_2_ were derived from industrial-scale data, while the Cu_2_O-coated P25-TiO_2_ used lab-scale data, using Cu_2_O NMs as a surrogate. Similar data uncertainty challenges were observed with the human and environmental toxicity data, where no experimental data were available, and the estimates for HTP and FEP were derived using read-across methods and available literature. For this reason, it is imperative that the data generated (environmental impact indicators and human and environmental toxicity data) are FAIR.

Uncertainties can be reduced by ensuring all NM safety data are FAIR and accessible from a centralized database, such as the European Union Observatory for Nanomaterials [66] or eNanoMapper [67]. Uncertainties can also be reduced by stimulating collaboration between material scientists (NM developers), toxicologists, and sustainability experts (LCA practitioners) to ensure the data are used optimally for SSbD applicability early in the innovation process. In this case, material scientists generate LCI data, toxicologists assess and identify human and environmental impacts, and sustainability experts combine the work of material scientists and toxicologists and use them to assess environmental impacts via LCA.

The framework is not based on a full LCA and only covers environmental sustainability aspects and human and environmental (nano)toxicity impacts. Therefore, its limitations fall under toxicological data limitations and availability of data for reading-across methods. If data are not available, then the framework is limited to the preliminary assessment—i.e., the concept design part—where KPIs can be calculated.

## 4. Conclusions

The purpose of this presented LCA framework is to identify environmental sustainability and toxicity hotspots early in the innovation process for future SSbD applicability. This prospective LCA framework allows for the comparison of a reference system (P25-titanium dioxide NMs) with a modified system (the Cu_2_O-coated/P25-TiO_2_) as an alternative with improved functionality. Additionally, an uncertainty heat map provides data gaps during framework application. Although there was a decrease in environmental impact (GWP and CED), the alternative had a relatively higher level of human and ecotoxicity (HTP and FEP). This case study illustrates the importance of performing an early safety and environmental sustainability assessment. For the presented case study, SSbD alternatives need to be considered that improve the photolytic activity but are not toxic to the environment. Overall, this prospective LCA framework was able to identify environmental sustainability and toxicity hotspots.

The application of the framework provided a first indicator of sustainability environmental and toxicity hotspots that can be taken into account in the design of NMs. Because of data scarcity, particularly for toxicological data and in emissions/disposal locations, effort needs to be put into reducing uncertainties, e.g., by producing more process knowledge, data generation, or prospective modelling.

This project was facilitated by an initiative from the Dutch Ministry of Infrastructure and Water Management, which provided a podium for this dialogue. Similar programs are needed for facilitating co-operation and collaboration between material scientists, and toxicologists/sustainability experts are needed to stimulate the operationalization of the SSbD concept in practice in a learning-by-doing approach.

## Figures and Tables

**Table 1:** Literature review of sustainability assessment frameworks for NMs and characteristics.

Reference	Type of Framework	Characteristics	LCA Consideration
[32]	LCA	A stepwise method to integrate LCA in each product development stage. This way, the output based on LCA for one stage of product development was input for the next stage of product development.	Yes
[20]	Sustainability assessment	A framework of 68 criteria for the sustainability assessment of nano-based products.	No
[33]	Toolkit to combine LCA with ternary diagrams	Safety is not considered except for LCA impact indicators, such as ecotoxicity and human toxicity. Toolkit development focused on applications on novel processes.	Yes
[34]	Early-stage life cycle screening of emerging technologies	Screening-to-LCA or a full LCA depending on data availability. The screening-to-LCA approach uses available data to evaluate the environmental performance of technologies at low TRLs.	Yes
[8]	Stepwise framework to improve sustainability and safety performance	The framework aims to guide the development of safer nano-based products at a laboratory-scale level and, when more information is available, more sustainable nano-based products at an industrial-scale level. Safety assessment precedes the sustainability assessment.	No

**Table 2:** Suggested KPIs inspired from green chemistry principles [35].

Indicator	Measure
Solvent consumption	Volume of solvent per nanomaterial mass (mL/g)
Electricity consumption	Amount per nanomaterial mass (kWh/g)
Heat consumption	Amount per nanomaterial mass (kJ/g)
Pollutant emissions	Mass of pollutants emitted per nanomaterial mass (g/g)
Waste production	Mass of waste produced per nanomaterial mass (g/g)

**Table 3:** Data needed for selected steps of system-level design of LCA.

Description	Data Needed	Alternative if Step CANNOT Be Performed
Step 6: Are the original system boundaries known?	Yes. Identification of LC stages	Estimation of KPIs
Step 7: What is the expected release rate of NM?	No. Research has to be identified for the NM under study	Build an LCI based on bulk material flows
Step 8: Do nano-toxicological data exist?	Yes, collection of effect factor (EF), human effect factor (HEF) and exposure factor (XF) for the NM under study	Use of nano-databases
Step 9: Data collection from sources such as the European Union Observatory for Nanomaterials (https://euon.echa.europa.eu/ (accessed on 10 September 2021))	Collection of effect factor (EF) and exposure factor (XF) for the NM under study	Use read-across method
Step 10a: Do fate data exist for the NM under study?	Yes. Fate factor (FF) data	Use read-across method
Step 10b: Is it possible to read across?	Yes. Psychochemical characteristics of NM	Build an LCI based on bulk material flows
Step 11: Data collection for LCI build-up	Material flows, nano-material flows and energy flows	Data for safety assessment or estimation of KPIs
Step 16: Scaling up	Good knowledge of thermodynamics and efficiencies for larger-scale equipment	None

**Table 4:** Physicochemical characteristics and toxicity characterization factors of considered NMs.

Nanomaterial	Particle Size (nm)	Surface Area (m^2^/g)	Characterization Factor _FEP_ (PAF.d.m^3^/kg_emitted_)	Characterization Factor _HTP_ (Cases.d/kg_emitted_)
P25-TiO_2_	20	50	3443 ^a^	222 ^b^
Cu_2_O/P25-TiO_2_	20 ^c^	50 ^d^	17,700 ^e^	0.99 ^b^

^a^ from [25,60], ^b^ from [27], ^c^ size growth after atomic layer deposition method is negligible, ^d^ expected but not measured, ^e^ from [38] FEP = freshwater eutrophication potential, HTP = human toxicity potential (non-cancer).

**Table 5:** LCA results per functional unit.

	Reference System (TiO_2_, Bulk-Based)	Reference System (P25-TiO_2_, Nano-Based)	Original System (Cu_2_O/TiO_2_, Bulk-Based)	Original System (Cu_2_O/P25-TiO_2_, Nano-Based)
GWP (kg CO_2_ eq.)	18.17	0	9.28	0
CED (MJ)	736.4	0	215.9	0
HTP_non-cancer_ (cases)	1.04 × 10^−6^	1.28 × 10^−2^	5.34 × 10^−6^	2.29 × 10^−5^
FEP (PAF.m^3^.d)	2632.3	15.08	9992.9	16.26

GWP, global warming potential; CED, cumulative energy demand; HTP, human toxicity (non-cancer) potential; FEP, freshwater ecotoxicity potential.

**Table 6:** Information needed for early toxicity analysis and LCA for SSbD applicability.

Parameter	Hotspots	Possible SbD Action to Relieve Hotspot
Size	Small NMs (<50 nm))	Alter design to avoid NMs below this threshold
Shape	High aspect ratio NMs (HARN, >1:5)	Alter design to avoid NMs with HARN
Solubility	Fibrous, non-soluble materials	Alter design to avoid fibrous, non-soluble materials
Stability of coating	Unstable coatings which allow for NM release	Alter design with stable coating
Persistence	Environmentally persistent	Alter design to avoid environmentally persistent NMs
Reactivity	Highly reactive NMs	Alter design to avoid reactive NMs
Reactive oxygen species (ROS)	Production of ROS and indirect genotoxicity	Alter design to reduce/avoid ROS production
Agglomeration	Agglomeration could be a potent inducer of inflammatory lung injury in humans	Alter design to NMs that do not agglomerate if lung exposure is expected
Exposure	Inhalation exposure (powders)	Avoid inhalation exposure
Environmental release rate	High NM release rate	Alter matrix design to avoid NM environmental release
Human toxicity indicator	High human toxicity from single process	Alter process to reduce human toxicity
Ecotoxicity indicator	High ecotoxicity from single process	Alter process to reduce ecotoxicity
Cumulative energy demand indicator	High energy consumption from single process	Alter process to reduce energy consumption
Any other environmental impact indicator	High contribution to indicator’s score by a single process	Alter process to reduce contribution to indicator

## Data Availability

Not applicable.

## Supplementary Materials

The following supporting information can be downloaded at: https://www.mdpi.com/article/10.3390/ijerph19074241/s1, Table S1: Data needed from nano-toxicity studies, Table S2: Life cycle inventory of reference system, Table S3: Life cycle inventory of additional processes for original system, Table S4: Calculation of characterization factors for freshwater ecotoxicity and human toxicity.
