# Supplementary material for: Safe-and-Sustainable-by-Design Framework Based on a Prospective Life Cycle Assessment: Lessons Learned from a Nano-Titanium Dioxide Case Study
Source: Int J Environ Res Public Health. 2022 Apr 2;19(7):4241. doi: 10.3390/ijerph19074241 (PMC8998679; doi:10.3390/ijerph19074241)
Supplement: Supplementary file 1 [file ijerph-19-04241-s001.zip › ijerph-1638281-supplementary.pdf]

Safe-and-sustainable-by-design framework based on a prospective life cycle assessment: lessons learned from a nano-titanium dioxide case study

Georgios Archimidis Tsalidis, Lya G. Soeteman-Hernandez, Cornelle W. Noorlander, Saeed Saedy, Ruud van Ommen, Martina G. Vijver, Gijsbert Korevaar

Supplementary Material

The Supplementary Material contains Tables which were omitted from the manuscript:

Table S1 presents descriptions and units for the Effect Factor, Human Effect Factor and Exposure factor of Step 8 of the framework.

Table S2 shows the Life Cycle Inventory of reference system

Table S3 shows the Life Cycle Inventory of additional processes for original system

Table S4 shows the calculation of characterization factors for freshwater ecotoxicity and human toxicity

Table S1. Data needed from nano-toxicity studies

Parameter	Description	Unit
Effect Factor (EF)	Hazardous concentrations of a substance at which 50% of the species are exposed to a concentration above their EC_{50} (i.e. concentration of a nanoparticle that gives half-maximal response).	$kg.m^{-3}$
Human Effect Factor (HEF)	Change in lifetime disease probability due to change in lifetime intake of a pollutant. It is calculated based on ED_{50} values which are extrapolated from animal in vivo tests.	$case.kg_{intake}^{-1}$
Exposure Factor (XF)	Dissolved fraction of NPs in an environmental compartment, e.g. aquatic environment. Occurring transformation processes are important, such as agglomeration.	dimensionless

Table S2. Life Cycle Inventory of reference system

**Input**			**Output**		
**TiO_2-NP production** [1]					
Electricity	2.85	kWh	TiO_2-NP	1	kg
V–Ti magnetite ore	5.71	kg	GWP	5.53	kg CO_2
Steel ball	0.001	kg			
Anthracite	10.076	MJ			
Coke	0.693	kg			
Liquid chlorine	0.25	kg			
Aluminum powder	0.06	kg			
Oxygen	0.693	kg			
Liquid caustic soda (30 %)	0.3	kg			
Saturated steam (1.3 MPa)	5.5	kg			
Petrol	0.017	kg			
Diesel	0.011	kg			
**Hydrogen production**					
TiO_2-NP	2.4	mg	H_2	63.012	µmol
Light (from lamp)	500	W	H_2	0.00012	g
Water	21.6	ml	Waste methanol (O_2 bound)	23.9998	g
Methanol	2.4	ml			
Catalyst	1	unit	Factor for 1 g H_2	7872.4	

Table S3. Life Cycle Inventory of additional processes for original system

**Input**			**Output**		
**Copper acetate** [2]					
Copper(II) carbonate	247.1098	kg	$Cu(CH_3COO)_2$	363.26	kg
Acetic acid	180.156	kg			
Sodium hydroxide	147.2	kg			
**Copper(II) hexafluoroacetylacetonate hydrate ($C_{10}H_4CuF_{12}O_5$)** [2]					
$Cu(CH_3COO)_2$	181.63	kg	$C_{10}H_4CuF_{12}O_5$	569.84	kg
Hexafluoroacetylacetone dihydrate	388.21	kg			
**Nanocoating with ALD**					
TiO_2-NP	1.5	g	TiO_2 coated with CuO	1.554484	g
$C_{10}H_4CuF_{12}O_5$	0.3395	g	CO_2	0.105096	g
Dionized water	5	L			
Pressurized nitrogen liquid	544.6952	g			
Cooling water		L			
Electricity for heating tapes	945	Wh			
**Hydrogen production**					
TiO_2 coated with CuO	2.484	mg	H_2	319.3335	µmol
Light (from lamp)	500	Wh	Waste methanol (O_2 bound)	23.99936	g
Water	21.6	ml			
Methanol	2.4	ml			

Table S4. Calculation of characterization factors for freshwater ecotoxicity and human toxicity

**Freshwater ecotoxicty**	P25	CuO	**Human toxicity**	P25	CuO
EF	26.9	7.025	EF	2.70	0.045
FF	128	2520	FF	130	22
XF	1	1	XF	1	1

References

1. Liao, W.; Heijungs, R.; Huppes, G. Thermodynamic Resource Indicators in LCA: A Case Study on the Titania Produced in Panzhihua City, Southwest China. *Int J Life Cycle Assess* **2012**, *17*, 951–961, doi:10.1007/s11367-012-0429-4.
2. Zhang, T.; Gu, H.; Ding, F.; Qu, F.; Dai, S. Synthesis, Characterization, and Thermostability of Bis(2,2,6,6-Tetramethyl-3, 5-Heptanedionato)Copper(II). *Rare Metals* **2012**, *31*, 343–349, doi:10.1007/s12598-012-0518-3.
